# SOCIAL INTEGRATION IN TEMPORARY HOUSING: PERSPECTIVES OF OLDER PERSONS EXPERIENCING HOMELESSNESS

**DOI:** 10.1093/geroni/igae098.1108

**Published:** 2024-12-31

**Authors:** Rachel Weldrick, Sarah Canham, Atiya Mahmood, Rachelle Patille, Shreemouna Gurung

**Affiliations:** McMaster University, Hamilton, Ontario, Canada; University of Utah, Salt Lake City, Utah, United States; Simon Fraser University, Vancouver, British Columbia, Canada; Simon Fraser University, vancouver, British Columbia, Canada; Simon Fraser University, Vancouver, British Columbia, Canada

## Abstract

The prevalence of homelessness among older persons in North America is rising. Accordingly, age-supportive housing and service models are needed to adequately support older persons experiencing homelessness to secure permanent housing. Within permanent supportive housing models, achieving and maintaining social integration (i.e., strong social connection and engagement) is recognized as a programmatic goal. Previous research has identified strategies to promote social integration in permanent supportive housing models, such as Housing First models. However, minimal research has considered strategies to promote social integration in temporary housing programs (THPs) and transitional housing models. This study addresses this knowledge gap by examining experiences of social integration, connection, and support in a THP program for older persons experiencing homelessness in Vancouver, Canada. We conducted semi-structured qualitative photovoice interviews with 11 current or former THP clients (Mean age = 65 years; 6 female, 5 male) across three sessions. Data were analyzed using a critical realist-informed thematic analysis method. We identified three themes: 1) technology access and programming can facilitate social connection; 2) consistent communication with program staff can enhance perceived social support; and 3) accessible built environments can promote social participation among clients. Findings offer insights for the development of age-supportive housing and support models for older persons experiencing homelessness. We conclude by offering strategies, such as digital literacy programming and the creation of ‘third spaces’, to support older persons to remain (or become) integrated while in temporary and transitional housing.

